# Elevated Levels of Environmental Enteric Dysfunction Biomarkers Among Rural Indonesian Infants: Associations with Water, Sanitation, Hygiene and Linear Growth

**DOI:** 10.3390/tropicalmed11090251

**Published:** 2026-09-07

**Authors:** Callum Lowe, Tony Arjuna, Mubasysyir Hasanbasri, Haribondhu Sarma, I Nyoman Sutarsa, Severine Navarro, Darren Gray, Matthew Kelly

**Affiliations:** 1 National Centre for Epidemiology and Population Health, College of Law, Governance, and Policy, Australian National University, Acton, ACT 2600, Australia; 2 Population Health Program, QIMR Berghofer Medical Research Institute, Brisbane, QLD 4006, Australia; 3 Faculty of Medicine, Public Health, and Nursing, Universitas Gadjah Mada, Yogyakarta 55281, Indonesia; tony.arjuna@ugm.ac.id (T.A.); mhasanbasri@ugm.ac.id (M.H.); 4 School of Medicine and Psychology, College of Science and Medicine, Australian National University, Acton, ACT 2601, Australia; sutarsa.nyoman@anu.edu.au; 5 Infection and Inflammation Program, QIMR Berghofer Medical Research Institute, Brisbane, QLD 4006, Australia

**Keywords:** environmental enteric dysfunction, sanitation, growth faltering, low- and middle-income countries, intestinal inflammation, unimproved water

## Abstract

**Objective:** To investigate the burden of environmental enteric dysfunction (EED) and its association with water, sanitation, and hygiene (WASH) and linear growth amongst infants in rural Central Java, Indonesia. **Study design:** A longitudinal study of 119 infants aged from 5–19 months was conducted in five villages of Wonosobo District, Central Java, Indonesia. Infant length and weight were measured at baseline and 5-month follow-up and stool samples were collected for the investigation of alpha-1-antitrypsin (AAT), neopterin (NEO), and myeloperoxidase (MPO) levels. Linear mixed-effects regression (LMER) models estimated the associations between WASH and height-for-age z-score (HAZ) on biomarker concentrations. **Results:** Biomarkers increased from baseline to follow-up where AAT, NEO, and MPO were elevated in 68.7%, 79.0%, and 71.4% of infants. Breastfed infants and infants with recent diarrhoea had higher biomarker levels than non-breastfed infants. After correction for multiple testing, few significant associations between EED biomarkers and WASH variables or HAZ were detected. EED biomarkers measured at baseline or follow-up were not significantly associated with growth faltering over the follow-up period. In cross-sectional analysis, compared to infants at risk of stunting (−2 < HAZ ≤ −1), infants with HAZ > −1 had lower AAT at baseline (β = −0.43, 95% CI = −0.82 to −0.05, p_adj_ < 0.05). **Conclusions:** Elevated EED biomarker levels were frequently observed and had mixed associations with WASH and HAZ, highlighting the complexity of EED and need to understand EED etiology through intervention studies.

## 1. Introduction

Poor water, sanitation, and hygiene (WASH) conditions are prevalent in low- and middle-income countries (LMICs) and remain a pervasive global issue [1]. Nearly one billion people practice open defecation and do not have access to improved water, resulting in chronic exposure to gastrointestinal pathogens with significant health and economic impacts in affected communities [1]. These conditions can lead to a vicious cycle of acute malnutrition and diarrhoea, the latter of which claims the lives of one million people, mainly children, annually [2]. Poor WASH conditions, in addition to non-infectious causes such as diets low in dietary diversity and animal-source foods, are also a major driver of chronic undernutrition, or stunting, which affects at least 149 million children under the age of five years globally [3,4]. Stunted children on average have reduced earnings as an adult [5], impaired cognitive development [6] and for girls increased risk of birth complications and the risk of giving birth to stunted children [7].

WASH interventions in LMICs have failed to demonstrate noticeable impacts on linear growth [8,9]. While many plausible reasons for this exist, including methodological issues such as poor intervention implementation, adherence and compliance, a major point of concern is the probability that household WASH interventions fail to break known and underrecognised exposure and transmission pathways that are unique to babies [10]. The degree of exposure is profound, with infants in low-resource settings demonstrated to have higher levels of faecal bacteria on their hands compared to their high-income counterparts [11]. High exposure to faecal bacteria has also been noted in infants’ complementary foods, on caregivers’ hands and play objects and in soil ingested (“geophagia”) as part of exploratory play behaviour [12]. This chronic, high exposure to faecal pathogens has been shown to lead to structural and physiological changes to the small intestine [13]. These changes, including but not limited to villous blunting, crypt hyperplasia, intestinal inflammation and translocation of bacterial products across a damaged intestinal barrier into systemic inflammation, have been referred to as environmental enteric dysfunction (EED) [14]. EED has been proposed as a missing link in understanding the causal chain between poor WASH conditions and linear growth faltering amongst infants in LMICs [15]. The lack of impact of household WASH infrastructure interventions on EED further supports the hypothesis that reducing chronic exposure to faecal pathogens in the environment that drive EED is critical to achieve reductions in stunting [16,17].

EED is increasingly viewed as a chronic mucosal adaptation to repeated early-life enteric pathogen exposure in settings of poverty, food insecurity and suboptimal WASH, rather than a single-pathogen syndrome [18]. Recurrent microbial and dietary insults drive epithelial stress and impaired barrier function (tight-junction disruption, altered production of mucus and antimicrobial peptides), with increased permeability and luminal antigen (pathogen-associated molecular patterns) access to the lamina propria [19]. This promotes sustained innate immune activation, such as neutrophils and activation of macrophages and monocytes, and downstream inflammatory remodelling of the small intestine [20]. Whilst histology of small intestine biopsy samples remains the gold standard to demonstrate epithelial injury with heightened mucosal activation to formally diagnose EED, serum, urine and stool biomarkers of one or more domains of EED have become the main approach to investigating EED [21]. Major strides have been made in identifying biomarkers of EED such as stool alpha-1-antitrypsin to assess protein loss/permeability, myeloperoxidase which is representative of neutrophil activation, neopterin for IFN-g associated macrophage activation and serum biomarkers such as lipopolysaccharide and endotoxin core antibody [22]. Some studies have found associations between these biomarkers and associated low-grade enteropathy which can reduce nutrient absorption and increase systemic inflammation likely contributing to poor linear growth and immune development amongst infants in LMICs [23,24]. There are some limited findings related to associations between EED biomarkers and infant-play behaviours such as geophagia amongst infants in Bangladesh [25,26] as well as close living in proximity to domesticated livestock [27]. Overall, however, the body of evidence linking infant-specific risk factors to EED is limited and there is a need for research to identify risk factors as they may be potential targets of “baby” WASH interventions [15]. In addition to this, the association between biomarkers of EED and linear growth is likely complex and non-linear [28]. Biomarkers may exhibit seasonality [29] and levels of biomarkers may be reduced due to downregulation of the immune system amongst infants who have experienced an exhaustive load of pathogen exposure [27,30].

Indonesia is a middle-income archipelago nation of 283 million residents, of which 100 million do not have access to improved sanitation and 117 million do not have access to improved water [31]. Regions in Indonesia with worse WASH conditions have higher rates of child stunting [32]. Whilst improvements have been made since 2018 in reducing child stunting, prevalence remains high in rural areas [33] and nationally was at 19.8% in 2024 [34]. Indonesia’s climate exhibits relatively definitive wet and dry seasons which may add an additional layer of complexity to understanding EED [29]. Stunting interventions in Indonesia are often limited to nutritional supplementation [35] which has marginal impact on linear growth [36]. There is little research on EED in Southeast Asia, particularly Indonesia [28]. However, existing research has investigated EED in poor areas where basic sanitation infrastructure and piped water are missing [37] as well as densely populated urban slums [38,39]. It remains relatively unexplored, however, as to whether EED is prevalent and/or a driver of stunting in communities that have basic WASH infrastructure but persistently high stunting rates. Considering these knowledge gaps and areas for further investigation, we conducted a longitudinal study of infants in rural Indonesia with the aim of measuring the association between WASH risk factors with EED biomarkers and linear growth faltering. We investigated EED at two distinct time points 5 months apart corresponding to dry and wet seasons to test an a priori hypothesis if associations between WASH, EED and linear growth differed by season.

## 2. Methods

This study was reported in line with the Strengthening the Reporting of Observational Studies in Epidemiology (STROBE) statement (Supplementary Table S1) [40].

### 2.1. Study Design, Sample and Population

This was a longitudinal observational study of rural infants from five villages in Wonosobo, Central Java, Indonesia. This is a mountainous area, and study villages were located 5–20 km from the Wonosobo town centre with household income predominantly arising from farming and livestock, small/micro businesses and manual labour jobs. Wonosobo is the district with the highest stunting prevalence in Central Java province [41].

Data were collected at two points—baseline data was collected during the dry season from August–September 2024, follow-up data was collected five months later during the wet season from January–February 2025. The wet and dry seasons in this mountainous area are not as contrasted as in other areas of Indonesia but are noticeable as fewer days of rain and lighter rain. The target age group for infants was six to eighteen months at baseline as this is a period of significant growth faltering [42] and all infants are expected to have begun weaning.

Infants were selected by multistage random sampling. Originally, two districts were selected in Wonosobo and a list of villages (“Desa”) across the two districts was obtained. Two villages in the smaller district and three villages in the larger district were randomly selected, and a list of all infants in each village combined with their sex, mother’s name, and household address was requested from district public health centres (“Puskesmas”). This list was narrowed down to infants who would be aged between 6 and 18 months at the time of planned baseline data collection. The number of infants recruited from each village was proportional to the total number of infants in each village.

Due to limited information on the central tendency and dispersion of EED biomarkers particularly amongst Indonesian infants, the sample size was determined with the Cohen’s d calculation as one sufficient to detect an a priori effect size of 0.5 standard deviations in EED biomarker concentrations between infants with HAZ < −1 and HAZ ≥ −1 with 5% probability of a type I error and 80% power which requires 128 infants. Due to restrictions on the size of ELISA test kits sold, the sample size had to be reduced to 120 which equates to a reduction in power from 80% to 77%.

Criteria for infants’ inclusion were that they were not suffering from a known chronic disease or severe infection requiring treatment (e.g., malaria, tuberculosis, dengue) and whose mother was willing to provide informed consent. Infants were excluded if their family had not been living in the current place of residence for the past 3 months or planned to move in the upcoming 6 months.

### 2.2. Data Collection

Prior to data collection, a training event was held to train data collectors on administration of the questionnaire, stool sample collection procedures, and anthropometric measurements of infants and mothers. Data collectors were local research assistants with prior experience in similar fieldwork. Data was regularly cross-checked between data collectors. Re-training of data collectors was performed prior to follow-up.

Data was collected via two household visits at baseline and at follow-up. Data collection at baseline involved a quantitative questionnaire with infants’ mothers using REDCap electronic data capture on tablets, anthropometric measurements of infants and their mothers, and stool sample collection. Follow-up data collection was similar except for mothers’ anthropometric measurements omitted and a shortened questionnaire only consisting of time-varying exposures and covariates. The questionnaire is available in the Supplementary Material.

The quantitative questionnaire pertained to household demographic and socio-economic factors, key maternal and paternal covariates, ownership of livestock, proximity of livestock to the household, infant’s health and diet, infant’s play activities, exposure to livestock, and key behaviours that may increase the risk of EED (e.g., frequency of geophagia, playing on a soiled surface). Further detail is provided in the exposures section. Consumption of animal-source foods (ASFs) was measured through a simple food frequency questionnaire as it is a key predictor of linear growth [43] and we theorise low intake may increase susceptibility to EED. Mothers were requested to respond with how frequently their infants had been consuming ASF: multiple times per day, daily, weekly, monthly, or never. These were added up across different categories to obtain the overall estimated daily serves of ASF.

Infant length was recorded using an infant length board (InnoQ Infantometer 100, PT Astra Components Indonesia, 1 mm graduation), and weight using a calibrated baby weight scale (InnoQ Digital Baby Scale 100, PT Astra Components Indonesia, 5 g graduation). For one participant, weight was measured as the average of two deducted weights (mother’s/local health cadre’s weight while cradling infant to chest minus own weight). Infants wore minimal clothing during weight measurement. Maternal height was measured using a portable stadiometer (InnoQ Stadiometer 100, 1 mm graduation) and weight with a standing weight scale (InnoQ Digital Flat Scale 100, 50 g graduation). No infants required their length (height) measured standing.

### 2.3. Stool Sample Collection and Analysis

At baseline and follow-up, mothers of infants were requested to assist in providing a non-diarrhoeal stool sample for the analysis of EED biomarkers. For infants who were experiencing diarrhoea at the time of data collection, mothers were requested to not collect any stool until infants’ faeces returned to normal texture and consistency. Mothers were provided with a stool sample collection container with an internally attached spatula, gloves, and zip-lock plastic and an additional plastic bag. Mothers were requested to collect a grape-sized amount of stool from infants’ diapers and to aim to collect stool from multiple areas in the diaper if possible. Stool samples were kept at 2–8 °C for <24 h before being transported to a local laboratory for storage at −20 °C for up to a month before being transported to a central laboratory in Jakarta and stored at −70 °C prior to biomarker analysis.

Stool samples were analysed to determine the concentration of faecal myeloperoxidase (MPO), neopterin (NEO), and alpha-1-antitrypsin (AAT). Analysis was performed using commercial ELISA kits according to the manufacturers’ instructions (AAT and MPO: Immundiagnostik AG, Bensheim, Germany, NEO: IBL Immuno-biological laboratories, Minneapolis, MN, USA). Samples were homogenized before analysis and were diluted 1:25,000 for AAT, 1:8 for NEO, and 1:500 for MPO. Produced in the liver, AAT is an enzyme inhibitor that protects the lungs from enzymes, however, its presence in the stool is an indicator of protein leakage in the intestinal tract [44], a hallmark of EED due to damage of the tight junctions between epithelial cells [45]. NEO is produced by macrophages after interferon-gamma stimulation, indicating intestinal immune activity and inflammation [46]. MPO is primarily produced by neutrophils and is involved in the production of antimicrobial reactive oxygen species and, therefore, it is an indicator of intestinal immune activity and inflammation. High MPO production is also damaging to normal intestinal tissue [47].

### 2.4. WASH Variables

A broad set of WASH exposures were considered in the quantitative questionnaire to capture multiple potential pathways of faecal pathogen exposure and important covariates. Two major categories were developed: (1) household WASH conditions (water source for cooking and drinking, improved sanitation, unsafe disposal of infant faeces (disposal into a river, fish pond, or outdoor bin), and presence of dirt floors in the household), (2) “babyWASH” potential pathogen exposure pathways: exposure to livestock faeces (poultry, cat, and bird faeces), caregiver WASH (storage of infant food at room temperature between feedings, mother washes hands with soap before preparing food, father handles animals and/or animal faeces), and play behaviour risks (infant regularly plays in gardens, infant regularly plays on roads, average daily time infant spends playing directly on soil/dirt/sand surfaces, frequency infant mouths soil/sand/dirt—“geophagia”, frequency infant mouths soiled or dirty fomites (e.g., outdoor play items, hand tools), frequency infant mouths animal faeces).

### 2.5. Statistical Analysis

#### 2.5.1. Data Preparation

Data cleaning was performed in IBM SPSS Version 26 [48] and all analyses were performed in RStudio (v2026.8.2) [49]. Anthropometry z-scores were computed in WHO AnthroPlus software (v1.0.4) [50] which has the 2006 WHO Child Growth Charts built in [51]. No outliers were detected using the cut-offs of +/−6 standard deviations [50]. Significant associations were considered as *p* < 0.05. EED biomarkers were natural log-transformed due to their skewed distribution. Whilst a subset of studies that have also analysed stool AAT, NEO, and MPO have aggregated these into a single “EED Score” (ref), we have elected not to do this as a recent systematic review [28] notes that evidence does not support this practice which falsely assumes that the direction of the association between these EED biomarkers and linear growth outcomes will always be positive.

#### 2.5.2. Descriptive and Bivariate Analysis

Descriptive analysis was performed to tabulate counts of infants across demographic, health, and WASH variables. The mean and standard deviation of anthropometric z-scores were calculated for baseline and follow-up. The median and interquartile range of EED biomarkers were calculated for baseline and follow-up and the proportion of observations at each time point that were elevated was calculated using the following cut-offs that were used in the MAL-ED study as well as other similar reports: AAT (<27 mg/dL), NEO (<70 nmol/L), MPO (<2000 ng/mL) [52]. Pearson correlations were computed for biomarkers within and between time points. Student’s *t*-tests were computed to compare the mean of log-transformed biomarkers at both time points individually across four key health variables: high ASF consumption (≥1.5 serves per day vs. <1.5 as a general proxy of higher consumption without detailed food volume data), recent 7-day diarrhoea, 3-month fever, and current breastfeeding status. Scatter plots of the age–biomarker relationship at each time point were overlayed with a simple linear model to visualise the impact of seasonality considering a hypothesized declining trend of the biomarkers with age. Kernel density plots were produced to visualize the raw distribution of biomarkers across boys and girls.

#### 2.5.3. Association Between WASH and EED Biomarkers

To account for repeated biomarker measurements within children and for clustering of children within villages, we fitted a linear mixed-effects regression (LMER) model with random intercepts for infant and village. This approach allowed pooling of baseline and follow-up data and estimating interactions with time. Although some predictors were time-invariant (e.g., SES characteristics), their effects on biomarker concentrations could vary over time. This was important to investigate because biomarkers showed low correlation within children across time points, indicating that both time-varying exposures and time-varying effects of stable predictors contribute to the observed variation. The models were developed in an iterative process:

Base mode: A null model with only random intercepts for infant and village was fitted to quantify the proportion of total variability attributed to clustering.

Step 1: A priori infant-level covariates were added: age, sex, time, current breastfeeding (yes/no), mother’s age, animal-source food intake, recent (7-day) diarrhoea, 3-month fever, and time of day of bowel motion of the stool sample provided. For current breastfeeding, animal-source food intake, diarrhoea, and fever, an interaction with time was added and significant interactions (*p* < 0.05) were retained. This set reflects key demographic and health determinants expected to influence biomarker concentrations.

Step 2 (Socio-economic model): Core SES confounders (mother’s highest education (senior secondary or higher vs. lower), household expenses (>Rp. 2,000,000 per month vs. below), and father’s occupation (employee/government vs. manual labour/farmer/other occupation) were included a priori regardless of statistical significance. Additional SES indicators (household bedroom count, ownership of livestock, wall material, and ownership of household assets including fridge, Wi-Fi, water heater) were included only if their main effect or time interaction reached *p* < 0.05. Average monthly household expenses were selected over household income as expenses are more reflective of long-term average income as month-to-month income can vary greatly in these rural areas.

Step 3 (WASH models): WASH variables were added into two separate models. The first model was a theory-based model only including household WASH variables (water source, household sanitation, presence of dirt floors in the household, and unsafe disposal of infant faeces). An interaction term with time was included if statistically significant. The second model consisted of the babyWASH variables including their interaction with time. To mitigate multicollinearity, babyWASH variables with a VIF > 5 in a full model were excluded before selection. Remaining babyWASH variables and their time interactions were selected using hybrid stepwise selection (entry/removal cut-off *p* < 0.01). This model was used to identify underrecognised babyWASH-EED pathways relevant to the local setting. To measure the effect of these variables on the EED biomarker level at baseline and follow-up separately, the emmeans package in R was used to extract the baseline and follow-up specific marginal effect from the time interaction term. To account for the testing of multiple outcomes, *p*-values were reported as crude and after adjustment using the Benjamini–Hochberg procedure, denoted as p_adj_. For linear mixed-effects models, model assumptions were evaluated through visual inspection of residual-versus-fitted plots and histograms of residuals to assess homoscedasticity and approximate normality.

#### 2.5.4. Association Between EED and Growth Faltering

The effect of EED on linear growth faltering was measured using ordinary least squares linear regression models with ∆HAZ as the dependent variable adjusting for baseline HAZ to account for regression to the mean. The models were adjusted for an a priori list of key covariates and confounders for linear growth including age, sex, breastfeeding status, birthweight, mother’s height, mother’s age, mother’s education, household expenses, father’s occupation, animal-source food intake, diarrhoea, fever, and significant WASH predictors of ∆HAZ. Models were fitted for baseline and follow-up biomarkers separately to avoid overfitting, and within each time point all biomarkers were modelled together to estimate their isolated effects on ∆HAZ. Baseline biomarkers regressed onto ∆HAZ reflect a temporal relationship, reflecting whether early EED predicted subsequent growth faltering. Linear regression assumptions including homoscedasticity and normality of residuals were assessed using residual diagnostic plots of residuals vs. fitted and histograms of residuals.

#### 2.5.5. Cross-Sectional Analysis Between HAZ and EED

To model how infants in different linear growth states differ in their biomarker concentrations, we modelled HAZ as an exposure in the same socio-economic-adjusted linear mixed-effects framework used for the WASH-EED analysis including significant WASH predictors of HAZ. We treated HAZ as a categorical variable to compare biomarker levels at both time points between infants with higher attained linear growth (HAZ > −1), infants at risk of stunting (−2 < HAZ ≤ −1) and infants who were stunted (HAZ ≤ −2). WHZ was included in all models to account for potential effects of acute malnutrition on biomarker concentrations. HAZ and WHZ were treated as time-varying predictors to model baseline HAZ/WHZ with baseline biomarkers and follow-up HAZ/WHZ with follow-up biomarkers. This represents the cross-sectional instantaneous association at each time point; that is, “What is the instantaneous association between an infant’s attained height-for-age or weight-for-height and their current state of intestinal inflammation and intestinal permeability?”. Time interactions were used to test whether the association differed significantly between baseline and follow-up. To account for the testing of multiple outcomes, *p*-values were reported as crude and after adjustment using the Benjamini–Hochberg procedure.

### 2.6. Ethics

This project received ethical approval from the Indonesian National Research and Innovation Agency (BRIN) Health Research Ethics Committee (Protocol #26032024004917) and the Australian National University Research Ethics Committee (Protocol H/2023/1123). Mothers provided informed consent for their participation in the research project after reading an information sheet outlining the project goals, involvement, risks and benefits. Mothers could withdraw from the project at any time with no implications.

## 3. Results

### 3.1. Descriptive Statistics of the Study Population

One hundred and twenty (120) participants were recruited at baseline with one lost to follow-up, leading to a total sample of 119 infants with non-diarrhoeal stool samples provided at baseline and follow-up. All proceeding analysis is presented for these 119 infants of whom all provided complete data. Participants were evenly split by sex (47.1% female) and across age groups (Table 1). Due to birthdate reporting errors in local data systems, infants’ age at baseline was between five and nineteen months instead of the intended six to eighteen months. Most infants were from households with average monthly expenses of 1–2 million rupiah (approx. $60–$120 USD) and approximately a third (32.8%) of households had average monthly expenses above 2 million rupiah. Just over a half of households (52.9%) owned livestock. Just above half of households owned a fridge (55.8%) and fewer owned Wi-Fi (38.3%) and fewer owned a water heater (21.7%). Most households (81.5%) had painted walls.

Common occupations of infants’ fathers were farmer (32.5%), manual labour jobs (23.3%) and owner of a small or micro business (16.7%). Mothers’ education was low with 32.8% of mothers reaching secondary high school or higher education. Most infants were currently being breastfed at baseline (90.8%) which only dropped slightly to 83.2% at follow-up. Approximately one third of infants (36.1%) were currently receiving formula milk at follow-up. About half of the infants consumed ≥ 1.5 serves of animal-sourced food per day at both baseline and follow-up. Seven-day diarrhoea prevalence increased from 10.9% at baseline to 13.4% at follow-up. Approximately half of mothers at baseline (51.3%) and follow-up (52.1%) reported their infant had a fever in the last three months. All participants were exclusively breastfed for the first 6 months of life.

Infants were born with an average birth weight of 3008 g and only three infants were wasted at follow-up. Stunting prevalence was slightly above a quarter (27.7%) at baseline but mean HAZ was low at −1.26 at baseline which decreased to −1.35 by follow-up, corresponding to an average ΔHAZ of −0.09. The loss of WAZ over the follow-up period was larger, dropping from −0.55 to −0.71 at follow-up which corresponded with a drop in WHZ from 0.15 at baseline to −0.09 at follow-up.

### 3.2. WASH Conditions and babyWASH

Overall, WASH conditions were mixed between participants households (Table 2). At the household level, WASH risk factors were prevalent with partial dirt floors (29.4% of households), unimproved sanitation (37.0% of households), and unsafe disposal of infant faeces (30.2% of households). Unimproved sanitation among sample households was exclusively toilets piped to a fishpond or river. Food and water risks were present with municipal piped tap water provided to only 29.4% of infants’ households while 70.6% relied on piped water from nearby springs, and infant foods were routinely stored at room temperature for prolonged periods between feedings by 49.6% of mothers. Livestock faeces exposure was not commonly reported with only 13.4% of infants exposed to poultry faeces and 21.0% exposed to bird faeces, although 40.3% were exposed to cat faeces and 36.7% of infants had a father who regularly handled livestock or livestock faeces. Additionally, 65.5% of mothers reported washing their hands with soap before preparing meals.

Mothers reported their infants to generally play and engage in exploratory behaviour in their environments which was more prevalent at follow-up. Mouthing of soil/sand/dirt, or geophagia, was practiced at least weekly by 32.7% of infants at follow-up which was an increase from 10.1% at baseline. More frequent, however, was the mouthing of soiled fomites which increased from 23.6% at least weekly at baseline to 56.3% at least weekly at follow-up. Mouthing of animal faeces was rare and only reported by the mothers of five infants at baseline. A smaller proportion of mothers reported their infants regularly played on nearby roads (24.4%) or in gardens (20.2%) at follow-up. The median of the average daily time infants spent playing on soiled surfaces as reported by mothers increased from 10 min at baseline to 30 min at follow-up.

### 3.3. Crude Biomarker Levels

EED biomarkers were elevated at baseline and follow-up in most infants (Table 3). The median (interquartile range) of AAT at baseline was 37.2 (21.4–70.7) mg/dL which decreased slightly to 35.7 (24.4–64.3) mg/dL. Median NEO level increased nearly 50% from 321.9 (135.6–661.4) nmol/L at baseline to 499.9 (106.0–1041.0) nmol/L at follow-up. Similarly, median MPO level increased from 2544 (1459–4276) ng/mL at baseline to 3883 (1842–12,590) ng/mL at follow-up. Nearly half of participants had elevated levels of AAT (45.4%) and MPO (47.9%) at both baseline and follow-up, while nearly two-thirds (67.2%) had elevated NEO at both baseline and follow-up. AAT and MPO were correlated moderately instantaneously at both timepoints (baseline: r = 0.40, *p* < 0.001 and follow-up: r = 0.34, *p* < 0.001) (Supplementary Table S2). There were no correlations in the same biomarker between timepoints, but baseline NEO was correlated with follow-up MPO (r = 0.24, *p* < 0.01). AAT and NEO exhibited circadian patterns (Supplementary Table S3) whereby stools taken during the daytime (6 a.m.–6 p.m.) had higher concentration of AAT (β = 0.67, 95% CI = 0.26 to 1.08. *p* < 0.01) and lower NEO (β = −0.52, 95% CI = −0.96 to −0.08, *p* < 0.05) compared to stools taken in the late evening or early morning. All multivariate analysis included adjustment for bowel motion time of day.

### 3.4. Biomarker Distributions Across Key Covariates

**Figure 1:**
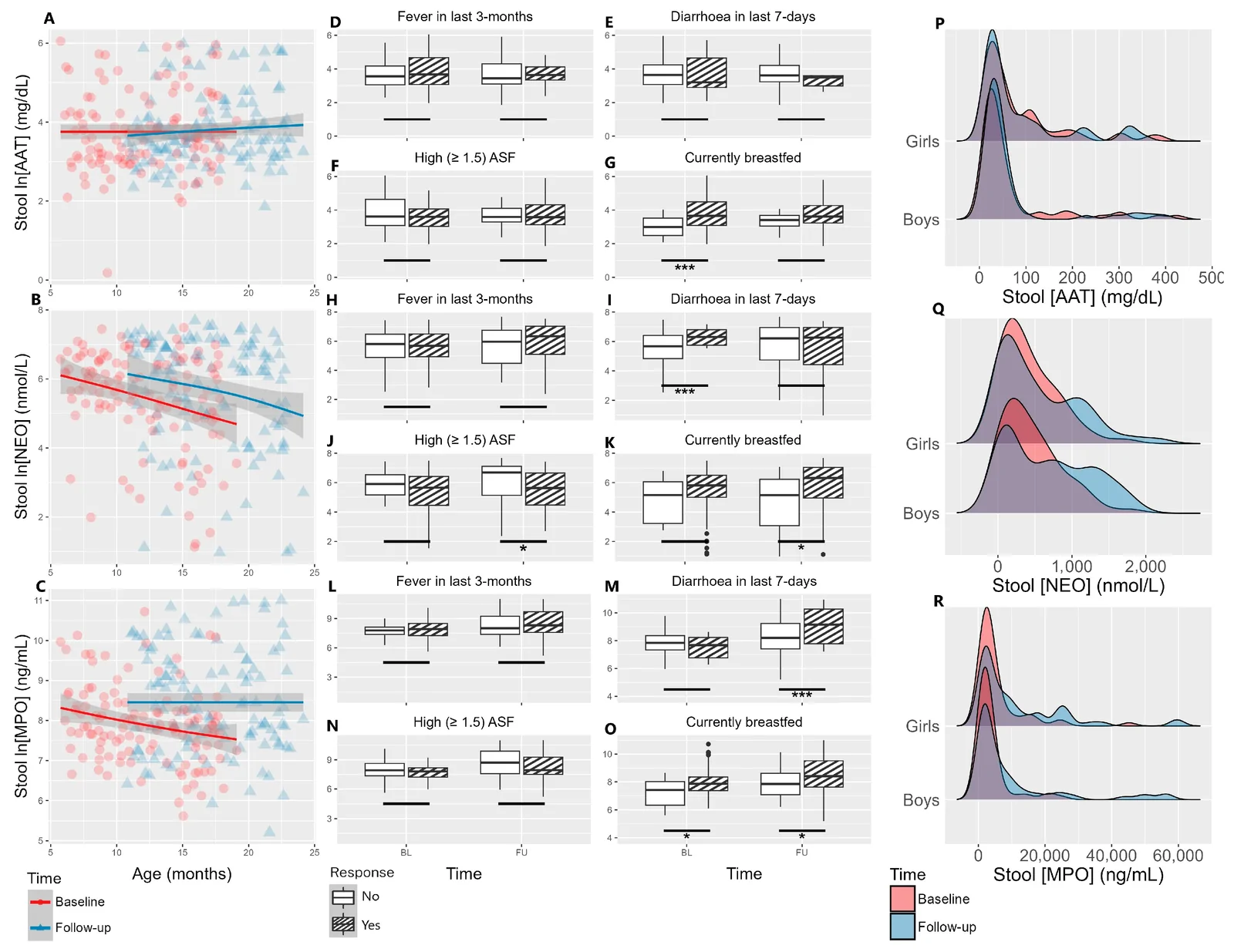
Distribution of EED biomarkers by age and sex, and key health covariates. (**A**–**C**): Scatter plots of log-transformed biomarkers across age at baseline (red circles) and follow-up (blue triangles). Shaded grey line represents confidence interval for a simple linear model of biomarker value regressed against age. (**D**–**O**): Boxplots of log-transformed biomarkers by key covariates—fever in last 3 months, diarrhoea in last 7 days, high animal-source food (ASF) intake, and current breastfeeding status, at baseline and follow-up. Shaded boxes represent a yes response. Asterisks represent *p*-values for a t-test comparing mean biomarker levels at each time point between the categories of the covariate response (no/yes). * *p* < 0.05 *** *p* < 0.001. (**P**–**R**): Kernel density plots of untransformed stool AAT, NEO, and MPO concentrations stratified by sex and time (red = baseline, blue = follow-up).

The distribution of biomarker values across key covariates is presented in Figure 1A–N. AAT levels at both time points were not correlated with age and showed little variation in their distribution over time. NEO showed a significant negative correlation with age at both time points (*p* = 0.004) and MPO showed a significant negative correlation with age at baseline only (*p* = 0.003). MPO and NEO, however, showed an upward shift to higher levels at follow-up across all ages, meaning the biomarker levels in 10–15-month-olds at follow-up were like infants 5 months younger at baseline. AAT and MPO levels were highly right-skewed, and NEO was moderately right-skewed. No significant differences in any biomarker level were observed between infants whose mother had or had not reported their infant having a fever in the last 3 months. Recent 7-day diarrhoea, however, was associated with elevated NEO at baseline (*p* < 0.001) but not follow-up and increased MPO at follow-up (*p* < 0.001) but not baseline. Infants who were consuming high (≥1.5 serves) ASF intake showed lower NEO at follow-up (*p* < 0.05) but no differences in AAT or MPO. Infants who were currently receiving breastmilk had raised mean biomarker values which were statistically significant for AAT at baseline (*p* < 0.001), NEO at follow-up (*p* < 0.05) and MPO at baseline (*p* < 0.05) and follow-up (*p* < 0.05).

### 3.5. Associations Between WASH and babyWASH with EED Biomarkers

Key household-level core WASH variables were weakly associated with biomarker concentrations (Table 4). There were no significant associations between household sanitation and biomarker levels. Whilst infants with a municipal water source trended towards having higher AAT, NEO, and MPO at follow-up, the *p*-values adjusted for multiple testing were insignificant. Infants in households that practiced unsafe disposal of infants’ faeces had significantly higher stool NEO at follow-up (β = 0.82, 95% CI = 0.20 to 1.43, p_adj_ < 0.05). Infants in households with partial dirt floors had significantly lower AAT on average, although the adjusted *p*-value was not statistically significant, but had significantly lower MPO on average (β = −0.59, 95% CI = −0.91 to −0.27, p_adj_ < 0.001). Hybrid stepwise selection of the babyWASH variables found they were very weakly associated with EED biomarkers. Infants whose complementary foods were stored at room temperature showed an increase in AAT over the follow-up and higher MPO on average, however, these associations were statistically insignificant after adjusting for multiple testing.

### 3.6. Association Between EED Biomarkers and Linear Growth Faltering

There were no significant associations between EED biomarkers measured at baseline or follow-up with linear growth faltering over the follow-up period (Table 5). Higher NEO concentration at follow-up was associated with ∆HAZ but not statistically significant (β = −0.04, 95% CI = −0.09 to 0.01, *p* = 0.09). Associations between all other biomarkers and ∆HAZ were weak and had *p*-values above 0.4.

### 3.7. Cross-Sectional and Associations Between EED and Anthropometry

The adjusted association between anthropometry and EED biomarkers revealed mixed results (Supplementary Table S4). Whilst a number of significant and strong associations were found between HAZ and the biomarker levels, these were not statistically significant after adjustment for multiple testing. An exception was that infants with HAZ > −1 compared to infants at risk of stunting at baseline had lower baseline AAT (β = −0.43, 95% CI = −0.82 to −0.05, p_adj_ < 0.05). Associations between HAZ and NEO were very weak with all crude *p*-values above 0.4. Whilst the association between MPO and HAZ at follow-up displayed an interesting non-linear U-shaped pattern when compared to infants at risk of stunting, stunted infants had higher MPO (β = 0.50, 95% CI = 0.02 to 0.99, *p* < 0.05) and infants with HAZ > −1 also had higher MPO (β = 0.59, 95% CI = 0.08 to 1.09, *p* < 0.05), the adjusted *p*-values were not statistically significant at p_adj_ = 0.09. Lower MPO at follow-up was also seen in infants with WHZ > 0 (β = −0.54, 95% CI = −0.93 to −0.15, p_adj_ < 0.05).

## 4. Discussion

This study demonstrated that infants in rural Central Java, Indonesia, a population with little to no prior EED research, are experiencing high levels of intestinal inflammation and protein loss and confirms the hypothesis that in this setting biomarkers of EED are both considerably elevated and associated with WASH conditions and anthropometry. Seasonality was observed in intestinal inflammation biomarkers MPO and NEO where mean concentrations were higher at follow-up (wet season) despite the older age of infants at follow-up and negative correlation between MPO and NEO with age. This contributes to some existing evidence [29,52] of the slight seasonality of EED biomarkers. Stool AAT and MPO levels at baseline and follow-up were similar to levels seen in children 0–5 years old in Bangladesh [53] and India [54] as well as multiple sites of the MAL-ED study [52] although the 75th percentile of MPO seen at follow-up (12,590 ng/mL) in our study was high. Median NEO levels were slightly lower but were still considered elevated in all but 17% of infants at baseline and were 20–50 times higher than in healthy and norovirus-infected children < 5 years old in urban Indonesia [55]. Baseline mean HAZ was already low (−1.26) and overall HAZ only declined by an average of −0.09. This may explain why biomarkers did not exhibit significant associations with ΔHAZ as little growth faltering over the follow-up occurred. Infants in our study may also be past an EED biomarker-sensitive period where biomarker levels predict subsequent linear growth (ΔHAZ). A number of other studies, however, have found significant associations between EED biomarkers with subsequent linear growth [28]. Only NEO at follow-up appeared to have a slight association with ΔHAZ although the association was not significant (*p* = 0.09).

EED biomarkers were weakly associated with WASH and babyWASH factors. Associations between spring water source (vs. municipal water) and biomarkers were moderate, associated with higher MPO at baseline but a decrease in MPO and a significant increase in NEO over the follow-up period (Table 3). Unsafe water source has been associated with increased intestinal permeability [56], but existing literature does not explain the shifts over time that we saw amongst our study population. It is difficult to juxtapose spring water as “unimproved” and municipal water as “improved” in this setting as, in Indonesia [57] and noted elsewhere [58], municipal piped water supplies are frequently contaminated. A novel finding from our study was the association between prolonged storage of complementary foods and elevated AAT and MPO; a risk factor that previously has been associated with diarrhoea [59] as well as elevated *E. coli* levels in complementary foods [60] although *p*-values increased to 0.10 after multiple testing adjustment. There was an indication that the practice of geophagia was associated with elevated MPO at follow-up (*p* < 0.05, p_adj_ = 0.10) which is consistent with other studies [25,26]. The lack of consistent associations between babyWASH factors may relate to measurement error and the influence of other factors that relate to EED biomarker levels.

The associations between attained HAZ and EED biomarkers were complex. Compared to infants at risk of stunting, those with the highest HAZ exhibited lower AAT at baseline, consistent with the theory that poorer growth is associated with epithelial barrier disruption and increased protein loss. Whilst MPO and HAZ showed no association at baseline, at follow-up the relationship was best described as U-shaped with higher MPO in both infants with the highest HAZ and those stunted at follow-up, compared with infants at risk of stunting. This may support the interpretation that MPO may reflect a proximal, episodic mucosal inflammatory biomarker, consistent with acute neutrophil recruitment and degranulation in response to recent enteric exposures, rather than cumulative chronic enteropathy. This is strengthened by the elevated MPO among infants with lower WHZ (acute undernutrition) at follow-up, linking MPO more closely to recent illness and acute undernutrition than to longer-term linear growth trajectories. The biological basis of the U-shaped relationship is unclear. It may reflect recent pathogen exposure accompanying infant increased mobility and environmental contact, whereas elevated MPO among stunted infants may reflect ongoing inflammatory burden, recurrent infections or altered mucosal function after barrier injury. Some studies have identified that elements of EED may be a survival advantage in the form of mucosal “containment” adaptation in the context of chronic pathogen exposure amongst infants with particularly poor linear growth [61]. Residual confounding may also be a possibility in the associations reported, including differential caregiving and exposure patterns where infants with better growth may subsequently have fewer restrictions on play (increasing exposure of contaminated surfaces), while caregivers may increase close-contact care and reduce environmental exposure for infants who have become stunted, a mechanism that could influence luminal neutrophilia (MPO) independently of chronic barrier permeability (AAT). The significant findings interpreted here should be taken with caution considering the inflation of *p*-values after multiple testing adjustment and treated as an area worth further exploration in larger studies.

An incidental finding of this analysis was that infants who were still breastfed had higher AAT, NEO, and MPO concentrations. This may reflect residual confounding whereby mothers who discontinued breastfeeding differ in hygiene practices, complementary food provision, and daily pathogen exposure patterns. It is unlikely that microquantities of biomarkers present in breastmilk “contaminate” stool samples as the concentrations are too small in comparison as also argued by McCormick and colleagues [52]. Rather, it is possible this is driven by genuine biological effects of breastmilk on mucosal immunity and microbial exposure, rather than a direct causal increase in enteropathy severity. Breastmilk can reshape the mucosal immune landscape and microbial ecology in ways that could shift biomarker readouts without necessarily worsening structural EED. Breastmilk delivers IgA, antimicrobial factors (e.g., lactoferrin, lysozyme), cytokines/growth factors, and human milk oligosaccharides, which collectively modulate microbial colonisation, epithelial barrier maturation, and immune education [62]. These inputs can alter the balance between immune “containment” and immune activation, such as changes in microbial exposure patterns and antigen sampling that may bias toward IFN-γ–linked myeloid activation (potentially increasing neopterin, a Th1-associated macrophage activation marker) even while improving certain barrier functions [63]. Accordingly, we interpret breastfeeding associations cautiously as potentially reflecting residual confounding and/or immunological programming effects and not necessarily a causal increase in enteropathy severity.

We found infants who had diarrhoea within the last week had significantly higher NEO at baseline and MPO at follow-up in their second non-diarrhoeal stool sample taken up to a week later (Supplementary Table S3). This is an interesting observation that future studies should also consider as studies that exclude infants with diarrhoea on the day of data collection and therefore do not include recent diarrhoea in their analysis [64] would fail to adjust for this significant contributor.

In this area of Indonesia, the main stunting programmes relate to complementary food supplementation and vaccination but not WASH, highlighting the need for “babyWASH” interventions [15]. Intensive WASH interventions including the WASH-Benefits and SHINE trials found no impact on linear growth, suggesting that the associations between WASH and linear growth may represent unmeasured confounding variables [8]. Our analysis did not find consistent associations between WASH, EED biomarkers, or linear growth, further highlighting the complexity of the WASH linear growth pathway.

The findings of our research should be interpreted carefully considering the small sample size. Linear growth of children is affected by a multitude of environmental, nutrition, infection, and genetic factors and explaining small deviations in linear growth with stool biomarkers in a small sample size may lead to the loss of statistical significance in small regression coefficients. The three biomarkers here represent important domains but not all domains of EED, and so readers should not assume that the findings presented here represent associations between WASH and growth with EED holistically. The variables measured in this dataset explained only ~30% of the variation in biomarker concentrations, highlighting the possibility of other unmeasured WASH and non-WASH factors influencing their concentration. Infants had a relatively low HAZ at baseline, therefore there may exist other factors such as poor infant and young child feeding practices or infections that had played a role in growth faltering amongst participants prior to their recruitment into this study. A larger cohort study recruiting infants prior to birth with a larger sample size is therefore warranted to better understand the complex associations presented here. As discussed, infants who are or become stunted likely share patterns of EED biomarkers over time rather than similar levels at a given time point. Overall, a holistic evaluation of the multitude of potential pathways leading to exposure of gastrointestinal pathogens that may trigger EED and growth faltering is required. We did not conduct repeat measurements of stool for biomarker measurement within the two time points, thus there may be day-to-day error unaccounted for.

In conclusion, our study identifies elevated levels of common faecal EED biomarkers amongst infants in a rural population of Indonesia. The overall associations between EED biomarkers and WASH and linear growth outcomes were generally weak and mixed, highlighting the need to better understand EED through intervention studies.

## Figures and Tables

**Table 1:** Demographic, socio-economic, health and anthropometry of the study sample.

**Demographic/Socio-Economic Variables**		**N (%)**	
Female sex		56 (47.1)	
Age (baseline)	5–12 months	60 (50.4)	
	12–19 months	59 (49.6)	
Household owns livestock		63 (52.9)	
Household average expenses per month	<Rp. 1 million	18 (15.0)	
	Rp. 1–2 million	62 (51.7)	
	>Rp. 2 million	40 (33.3)	
Mother highest education	Primary	38 (31.9)	
	Middle school	42 (35.3)	
	Secondary or higher	39 (32.8)	
Household bedroom count	1–2	79 (66.4)	
	≥3	40 (33.6)	
Household has finished (painted) walls		97 (81.5)	
Father’s occupation	Farmer	39 (32.5)	
	Manual labour	28 (23.3)	
	Small/micro business	20 (16.7)	
	Employee/other	31 (27.5)	
Household owns Wi-Fi		46 (38.3)	
Household owns a water heater		26 (21.7)	
Household owns a fridge		67 (55.8)	
Mother’s age (median, 25th, 75th percentile)		28 (25, 32)	
**Infant Health ^2^**		**Baseline**	**Follow-Up**
Currently breastfed		108 (90.8)	99 (83.2)
Currently formula-fed		20 (16.8)	43 (36.1)
Consume ≥ 1.5 serves ASF/day ^1^		56 (47.1)	67 (56.3)
Diarrhoea in last 7 days		13 (10.9)	16 (13.4)
Fever in last 3 months		61 (51.3)	62 (52.1)
**Anthropometry ^2^**		**Baseline**	**Follow-Up**
Weight-for-height z-score (WHZ) (mean ± SD)		0.15 ± 1.01	−0.09 ± 0.98
Weight-for-age z-score (WAZ) (mean ± SD)		−0.55 ± 1.00	−0.71 ± 0.97
Height-for-age z-score (HAZ) (mean ± SD)		−1.26 ± 1.02	−1.35 ± 0.95
Stunting prevalence N (%)		33 (27.7)	24 (20.2)
Wasting prevalence N (%)		2 (1.7)	3 (2.5)
Birthweight (Mean ± SD) ^3^		3.008 ± 0.430 kg	

Diarrhoea and fever were self-reported by infants’ mothers. Stunting defined as HAZ ≤ −2, wasting as WHZ ≤ −2. ^1^ Consumption of animal source foods (ASFs) obtained from a food frequency questionnaire. ^2^ Infant health and anthropometry were measured at baseline and at follow-up while demographic and socio-economic variables were recorded at baseline only. ^3^ Birthweights were recorded from local vital health data books.

**Table 2:** WASH and babyWASH conditions in the study sample.

**Household WASH Conditions ^1^**	**N (%)**	
Spring water source (vs. municipal)	84 (70.6)	
Unimproved sanitation	44 (37.0)	
Infant faeces disposed of unsafely	36 (30.2)	
Partial dirt/earth floors (vs. fully cemented/tiled)	35 (29.4)	
Father handles livestock or livestock faeces	44 (36.7)	
**Mother’s WASH ^1^**		
Mother washes hands with soap before preparing meals	78 (65.5)	
Infants’ foods are stored at room temperature for prolonged periods	59 (49.6)	
**Exposure to Livestock**	**Baseline**	**Follow-Up**
Exposed to poultry faeces	10 (8.4)	16 (13.4)
Exposed to bird faeces	16 (13.4)	25 (21.0)
Exposed to cat faeces	35 (29.4)	48 (40.3)
**Play Behaviour**		
Mouth soil/sand/dirt at least weekly	12 (10.1)	39 (32.8)
Mouth soiled fomites at least weekly	28 (23.5)	67 (56.3)
Ever mouth animal faeces (vs. never)	5 (4.2)	5 (4.2)
Average daily play time on soiled surfaces ^2^	10 (0, 35)	30 (30, 60)
Infant plays on nearby roads	27 (22.6)	29 (24.4)
Infant plays in nearby gardens	22 (18.5)	24 (20.2)

Data expressed as N (%). ^1^ Merged cells are for time-independent variables captured in the baseline questionnaire only. ^2^ Data expressed as median (25th, 75th percentile).

**Table 3:** Median and interquartile ranges of untransformed biomarker values at baseline and follow-up and proportion of observations elevated in comparison to reference levels.

	AAT mg/dL		NEO nmol/L		MPO ng/mL	
	Baseline	Follow-Up	Baseline	Follow-Up	Baseline	Follow-Up
Median	37.2	35.7	321.9	499.9	2544	3883
Interquartile range	21.4–70.7	24.4–64.3	135.6–661.4	106.0–1041	1459–4276	1842–12,590
Elevated concentrations ^1^ N (%)	75 (63.0)	81 (68.7)	99 (83.2)	94 (79.0)	79 (66.4)	85 (71.4)
Elevated both time points	54 (45.4)		80 (67.2)		57 (47.9)	

^1^ Reference concentrations derived from McCormick et al. (2017) as follows: AAT (<27 mg/dL), NEO (<70 nmol/L), MPO (<2000 ng/mL) [52].

**Table 4:** Linear mixed-effects regression model of the associations between WASH variables and natural log-transformed EED biomarkers. All models are adjusted for age, sex, breastfeeding status, mother’s age, animal-source food intake, recent diarrhoea, fever, bowel motion time of day, and socio-economic variables. Baseline and follow-up isolated effects are modelled when the interaction with time is significant at *p* < 0.10. Random intercepts are included at the infant and village levels to take into account clustering and repeated measures.

	Average Effect ^1^			Interaction with Time			Effect at Baseline ^3^			Effect at Follow-Up ^3^		
	β (95% CI)	*p*	p_adj_	β (95% CI)	*p*	p_adj_	β (95% CI)	*p*	p_adj_	β (95% CI)	*p*	p_adj_
AAT												
Model 1. Household WASH variables												
Unimproved sanitation	0.21 (− 0.05, 0.47)	0.11	0.31	0.16 (−0.30, 0.62)	0.5	0.78	0.13 (−0.27, 0.53)	0.51	0.78	0.29 (−0.11, 0.69)	0.15	0.29
Municipal water (vs. spring)	0.15 (−0.14, 0.44)	0.31	0.59	0.41 (−0.10, 0.92)	0.11	0.31	−0.05 (−0.45, 0.34)	0.79	0.89	0.36 (−0.04, 0.76)	0.08	0.37
Unsafe disposal of infant faeces	0.15 (−0.13, 0.44)	0.28	0.57	−0.07 (−0.57, 0.43)	0.77	0.89	0.19 (−0.2-, 0.58)	0.33	0.61	0.12 (−0.27, 0.50)	0.55	0.81
Partial dirt floors in household	−0.29 (−0.57, −0.00)	0.046	0.10	−0.17 (−0.67, 0.34)	0.51	0.77	−0.20 (−0.58, 0.18)	0.29	0.57	−0.37 (−0.75, 0.01)	0.05	0.19
Model 2. Stepwise selected babyWASH variables ^2^												
Infant exposed to cat faeces	0.16 (−0.11, 0.43)	0.23	0.51	0.65 (0.19, 1.10)	0.005	0.021	−0.22 (−0.62, 0.17)	0.28	0.57	0.36 (0.03, 0.69)	0.032	0.10
Average daily time playing on soiled surfaces > 30 min	0.11 (0.01, 0.21)	0.031	0.10	0.04 (−0.20, 0.28)	0.76	0.89	−			−		
Infant’s foods are stored at room temperature between feedings	0.03 (−0.19, 0.25)	0.8	0.89	0.45 (0.02, 0.88)	0.040	0.10	−0.19 (−0.52, 0.14)	0.24	0.52	0.25 (−0.04, 0.54)	0.1	0.30
NEO												
Model 1. Household WASH variables												
Unimproved sanitation	0.11 (−0.39, 0.61)	0.63	0.83	0.28 (−0.51, 1.07)	0.49	0.77	0.02 (−0.73, 0.77)	0.95	0.98	−0.21 (−0.96, 0.54)	0.57	0.81
Municipal water (vs. spring)	0.32 (−0.15, 0.80)	0.18	0.45	0.67 (−0.18, 1.51)	0.12	0.32	−0.07 (−0.73, 0.59)	0.84	0.91	0.71 (0.05, 1.37)	0.035	0.10
Unsafe disposal of infant faeces	0.29 (−0.16, 0.75)	0.21	0.49	1.05 (0.23, 1.86)	0.012	0.12	−0.23 (−0.84, 0.38)	0.45	0.77	0.82 (0.20, 1.43)	0.009	0.012
Partial dirt floors in household	0.02 (−0.42, 0.46)	0.93	0.98	−0.31 (−1.09, 0.46)	0.43	0.76	0.16 (−0.42, 0.75)	0.59	0.82	−0.13 (−0.70, 0.44)	0.66	0.85
Model 2. Stepwise selected babyWASH variables ^2^												
Infant mouths soiled fomites at least weekly	0.55 (0.10, 1.00)	0.017	0.12	0.14 (−0.32, 0.59)	0.53	0.79						
Mother regularly washes hands with soap before preparing meals	−0.39 (−0.74, −0.04)	0.029	0.13	−0.07 (−0.79, 0.66)	0.84	0.91						
MPO												
Model 1. Household WASH variables												
Unimproved sanitation	0.04 (−0.25, 0.34)	0.78	0.86	−0.04 (−0.59, 0.51)	0.94	0.98	0.10 (−0.36, 0.55)	0.67	0.85	−0.001 (−0.46, 0.46)	0.99	0.99
Municipal water (vs. spring)	0.08 (−0.25, 0.41)	0.63	0.83	0.94 (0.37, 1.5)	0.001	<0.05	−0.38 (−0.83, 0.07)	0.09	0.29	0.56 (0.11, 1.02)	0.016	0.11
Unsafe disposal of infant faeces	−0.06 (−0.38, 0.27)	0.74	0.89	−0.12 (−0.71, 0.47)	0.69	0.86	0.002 (−0.44, 0.44)	0.99	0.99	−0.11 (−0.54, 0.32)	0.62	0.83
Partial dirt floors in household	−0.59 (−0.91, −0.27)	<0.001	<0.001	0.40 (−0.20, 1.00)	0.19	0.45	−0.79 (−1.23, −0.36)	<0.001	<0.001	−0.40 (−0.82, 0.03)	0.06	0.21
Model 2. Stepwise selected babyWASH variables ^2^												
Infant’s foods are stored at room temperature between feedings	0.31 (0.04, 0.58)	0.024	0.10									
Infant mouths soil/sand/dirt at least weekly	0.16 (−0.17, 0.49)	0.34	0.62	0.54 (0.05, 1.03)	0.031	0.10	−0.13 (−0.48, 0.22)	0.47	0.78	0.41 (0.08, 0.74)	0.015	0.12

^1^ The average effect refers to the mean effect on AAT across both baseline and follow-up. ^2^ Stepwise selected babyWASH variables do not include the core WASH variables and are selected from an array of variables less established in the literature as posing a WASH-EED risk, including: exposure to poultry, cat, bird faeces, open storage of infant’s foods at room temperature, average daily time infant plays on ground, mother washes hands before preparing meals, mouthing of soil/sand/dirt, mouthing of soiled fomites, and father handles livestock/livestock faeces. AIC, Akaike information criterion. ICC, inter-class correlation. ^3^ Effect at baseline refers to the instantaneous association between the WASH variable measured at baseline and EED biomarker measured at baseline. The follow-up effect refers to the association between the household WASH variables measured at baseline or babyWASH variables measured at follow-up and EED biomarkers measured at follow-up. AIC: AAT model 1: 699.5, model 2: 694.3. NEO model 1: 892.3, model 2: 890.1. MPO model 1: 759.3, model 2: 756.4. R2: AAT model 1: 0.25, model 2: 0.34. NEO model 1: 0.26, model 2: 0.28. MPO model 1: 0.32, model 2: 2.7%. ICC: AAT model 1: 3.2%, model 2: 6.9%. NEO model 1: 4.4%, model 2: 4.8%. MPO model 1: 0.33, model 2: 6.6%. P_adj_ are Benjamini–Hochberg corrected *p*-values for multiple testing.

**Table 5:** Linear regression model of the association between EED biomarkers and change in growth (ΔHAZ) over the follow-up period. Model is adjusted for baseline HAZ, age, sex, breastfeeding status, mother’s height, mother’s education, household expenses, diarrhoea, fever, WASH, and animal-source food intake. Each biomarker is modelled in mutual adjustment with other biomarkers, but a separate model is fitted for each time point. Biomarkers are natural log-transformed from their original units: AAT (mg/dL), NEO (nmol/L), MPO (ng/mL).

		Association with ∆HAZ	
		β (95% CI)	*p*
AAT	Baseline	0.01 (−0.07, 0.09)	0.76
	Follow-up	0.02 (−0.07, 0.11)	0.68
NEO	Baseline	−0.01 (−0.07, 0.05)	0.69
	Follow-up	−0.04 (−0.09, 0.01)	0.09
MPO	Baseline	0.04 (−0.06, 0.13)	0.45
	Follow-up	0.02 (−0.04, 0.08)	0.51

## Data Availability

Data described in the manuscript will be made available upon reasonable request to the corresponding author.

## Supplementary Materials

The following supporting information can be downloaded at: https://www.mdpi.com/article/10.3390/tropicalmed11090251/s1, Supplementary Table S1. STROBE Statement—Checklist of items that should be included in reports of cross-sectional studies; Supplementary Table S2. Pearson correlation between and within baseline and follow−up biomarkers; Supplementary Table S3. Linear mixed effects regression modelling of the effects of demographic, socio-economic status (SES), and key covariates on log-transformed EED biomarker concentrations; Supplementary Table S4. Linear mixed-effects regression (LMER) modelling of the cross-sectional association between anthropometry and log-transformed EED biomarkers.

## Abbreviations

AAT; alpha-1-antitrypsin, ASF; animal-source food, EED; environmental enteric dysfunction, HAZ; height-for-age z-score, LMER; linear mixed-effects regression, LMICs; low- and middle-income countries, NEO; neopterin, MPO; myeloperoxidase, SES; socio-economic status, WASH; water, sanitation and hygiene, WHZ; weight-for-height z-score.
