# Supplementary material for: Elevated Levels of Environmental Enteric Dysfunction Biomarkers Among Rural Indonesian Infants: Associations with Water, Sanitation, Hygiene and Linear Growth
Source: Trop Med Infect Dis. 2026 Sep 7;11(9):251. doi: 10.3390/tropicalmed11090251 (PMC13611734; doi:10.3390/tropicalmed11090251)
Supplement: Supplementary file 1 [file tropicalmed-11-00251-s001.zip › tropicalmed-4471864-supplementary.pdf]

Supplementary material to: Elevated Levels of Environmental Enteric Dysfunction Biomarkers Among Rural Indonesian Infants: Associations with Water, Sanitation, Hygiene and Linear Growth

Supplementary Table S1. STROBE Statement—Checklist of items that should be included in reports of cross-sectional studies.

	**Item No**	**Recommendation**	**Page #**
**Title and abstract**	1	(*a*) Indicate the study's design with a commonly used term in the title or the abstract	1
		(*b*) Provide in the abstract an informative and balanced summary of what was done and what was found	2
**Introduction**			
Background/rationale	2	Explain the scientific background and rationale for the investigation being reported	3
Objectives	3	State specific objectives, including any prespecified hypotheses	4-5
**Methods**			
Study design	4	Present key elements of study design early in the paper	6
Setting	5	Describe the setting, locations, and relevant dates, including periods of recruitment, exposure, follow-up, and data collection	6-7
Participants	6	(*a*) Give the eligibility criteria, and the sources and methods of selection of participants	6-7
Variables	7	Clearly define all outcomes, exposures, predictors, potential confounders, and effect modifiers. Give diagnostic criteria, if applicable	7-8
Data sources/ measurement	8*	For each variable of interest, give sources of data and details of methods of assessment (measurement). Describe comparability of assessment methods if there is more than one group	*7-8*
Bias	9	Describe any efforts to address potential sources of bias	n/a
Study size	10	Explain how the study size was arrived at	6
Quantitative variables	11	Explain how quantitative variables were handled in the analyses. If applicable, describe which groupings were chosen and why	8-9
Statistical methods	12	(*a*) Describe all statistical methods, including those used to control for confounding	8-11

		(*b*) Describe any methods used to examine subgroups and interactions	8-11
		(*c*) Explain how missing data were addressed	n/a
		(*d*) If applicable, describe analytical methods taking account of sampling strategy	8-11
		(*e*) Describe any sensitivity analyses	11
**Results**			
Participants	13*	(a) Report numbers of individuals at each stage of study—eg numbers potentially eligible, examined for eligibility, confirmed eligible, included in the study, completing follow-up, and analysed	12
		(b) Give reasons for non-participation at each stage	n/a
		(c) Consider use of a flow diagram	n/a
Descriptive data	14*	(a) Give characteristics of study participants (eg demographic, clinical, social) and information on exposures and potential confounders	12
		(b) Indicate number of participants with missing data for each variable of interest	n/a
Outcome data	15*	Report numbers of outcome events or summary measures	13-14
Main results	16	(*a*) Give unadjusted estimates and, if applicable, confounder-adjusted estimates and their precision (eg, 95% confidence interval). Make clear which confounders were adjusted for and why they were included	14-17
		(*b*) Report category boundaries when continuous variables were categorized	14-17
		(*c*) If relevant, consider translating estimates of relative risk into absolute risk for a meaningful time period	n/a
Other analyses	17	Report other analyses done—eg analyses of subgroups and interactions, and sensitivity analyses	14-17
**Discussion**			
Key results	18	Summarise key results with reference to study objectives	18
Limitations	19	Discuss limitations of the study, taking into account sources of potential bias or imprecision. Discuss both direction and magnitude of any potential bias	22
Interpretation	20	Give a cautious overall interpretation of results considering objectives, limitations, multiplicity of analyses, results from similar studies, and other relevant evidence	19-22

Generalisability	21	Discuss the generalisability (external validity) of the study results	22
**Other information**			
Funding	22	Give the source of funding and the role of the funders for the present study and, if applicable, for the original study on which the present article is based	1

Supplementary Table S2. Pearson correlation between and within baseline and follow−up biomarkers. * p<0.05 ** p<0.01 *** p<0.001

	AAT_{BL}	NEO_{BL}	MPO_{BL}	AAT_{FU}	NEO_{FU}	MPO_{FU}
AAT_{BL}	1					
NEO_{BL}	−0.01	1				
MPO_{BL}	**0.40*** **	0.16	1			
AAT_{FU}	0.16	0.05	0.12	1		
NEO_{FU}	−0.04	0.14	0.15	0.03	1	
MPO_{FU}	0.08	**0.24** **	0.09	**0.34*** **	0.03	1

	ln[AAT] mg/dL				ln[NEO] nmol/L				ln[MPO] ng/mL			
	Average effect		Interaction with time		Average effect		Interaction with time		Average effect		Interaction with time	
	B (95% CI)	p	B (95% CI)	p	B (95% CI)	p	B (95% CI)	p	B (95% CI)	p	B (95% CI)	p
**Null model**	n/a		n/a		n/a		n/a		n/a		n/a	
Model fit												
R-squared	0/0.165				0/0.138				0/0.026			
ICC (individual	15.60%				12.80%				2.60%			
ICC (village)	0.10%				0.10%				0			
AIC	670.4				882.9				760.3			
**Base model**												
Age	**0.04 (0.02, 0.08)**	**<0.001**	ns		**-0.07 (-0.13, -0.01)**	**0.031**	ns		-0.01 (-0.05, 0.03)	0.66	ns	
Sex	**0.30 (0.05, 0.55)**	**0.001**	ns		-0.17 (-0.54, 0.20)	0.35	ns		**0.43 (0.13, 0.72)**	**0.001**	ns	
Currently breastfed	**0.51 (0.13, 0.89)**	**0.004**	ns		**0.94 (0.38, 1.50)**	**0.007**	ns		**0.55 (0.12, 0.98)**	**0.015**	ns	
ASF intake (serves per day)	-0.10 (-0.22, 0.02)	0.10	ns		-0.16 (-0.34, 0.02)	0.07	ns		**-0.17 (-0.30, -0.03)**	**0.031**	ns	
Mother's age	-0.02 (-0.04, 0.00)	0.07	ns		-0.003 (-0.04, 0.03)	0.86	ns		**-0.04 (-0.07, -0.01)**	**0.038**	**0.07 (0.02, 0.12)**	**0.006**
7-day diarrhoea	-0.13 (-0.49, 0.24)	0.50	ns		**1.04 (0.21, 1.86)**	**0.019**	**-1.39 (-2.5, -0.26)**	**0.013**	-0.25 (-0.87, 0.38)	0.43	**1.03 (0.19, 1.88)**	**0.005**
Daytime stool (vs. nighttime)	**0.67 (0.26, 1.08)**	**0.006**	**-0.78 (-1.36, -0.20)**	**0.005**	**-0.52 (-0.96, -0.08)**	**0.041**	ns		0.01 (-0.33, 0.35)	0.96	ns	
Fever in last 3-months	0.19 (-0.05, 0.43)	0.12	ns		0.14 (-0.23, 0.51)	0.46	ns		0.18 (-0.10, 0.46)	0.20	ns	
Model fit												
R-squared	0.14/0.21				0.17/0.17				0.20/0.23			
ICC (individual	8.70%				0%				4.70%			
ICC (village)	0.40%				0.50%				0%			
AIC	686.6				881.6				760.6			
**SES-adjusted model**												
Core variables												
Mother has high education[1]	-0.13 (-0.41, 0.16)	0.38	ns		0.10 (-0.50, 0.70)	0.74	ns		0.08 (-0.25, 0.41)	0.64	ns	
High household expenses[2]	0.27 (-0.01, 0.54)	0.06	ns		0.23 (-0.19, 0.65)	0.28	ns		0.41 (-0.03, 0.84)	0.07	**-1.00 (-1.58, -0.42)**	**<0.001**
Father's job[3]	-0.28 (-0.59, 0.02)	0.07	ns		0.06 (-0.43, 0.55)	0.81	ns		-0.07 (-0.55, 0.40)	0.76	**0.75 (0.12, 1.38)**	**0.020**
Other SES indicators												
High bedroom count	ns				ns				ns			
Own livestock	ns				ns				ns			
Finished walls	ns				ns				ns			
Own Wi-Fi	**0.30 (0.04, 0.56)**	**0.042**	ns		ns				ns			
Own water heater	ns				ns				ns			
Own fridge	0.26 (-0.09, 0.61)	0.15	**-0.37 (-0.67, -0.07)**	**0.021**	ns				0.04 (-0.37, 0.45)	0.84	**-0.55 (-1.10, -0.00)**	**0.049**
Model fit												
R-squared	0.25				0.25				0.31			
ICC (individual	6.20%				4.80%				6.60%			
ICC (village)	0				0.27%				0			
AIC	693.0				882.8				757.6			

Supplementary Table S3. Linear mixed effects regression modelling of the effects of demographic, socio-economic status (SES), and key covariates on log-transformed EED biomarker concentrations. All models include a random intercept for infant and for village in order to take into account clustering and repeat biomarker measures. R-squared values are written as the marginal/conditional variance explained. ICC; intra-class correlation at the individual and village level. AIC; Akaike information criterion. The SES-adjusted model includes all base model covariates. ns; not significant. Average effect refers to the mean effect of the covariate on baseline and follow-up biomarker levels in the pooled dataset.

[1]High education defined as Secondary or higher

[2]High household expenses defined as > Rp. 2.000.000 per month

[3]Fathers job where the exposure is employee or government employee and the reference is farmer, manual labour, or other occupation.

Supplementary Table S4. Linear mixed-effects regression (LMER) modelling of the cross-sectional association between anthropometry and log-transformed EED biomarkers. The LMER is an extension of the socio-economic and key covariate adjusted LMER used in the measurement of the association between WASH and EED with random intercepts for infant and village to account for clustering and repeated measurements. HAZ and WHZ are modelled jointly.

[1]The average effect refers to the mean association between the predictor and EED biomarkers across both time points.

[2]Effect at baseline refers to the association between HAZ and WHZ at baseline with EED biomarkers measured at baseline, effect at follow-up is the association between HAZ and WHZ at follow-up with EED biomarkers measured at follow-up.

[3]p_{adj} is the Benjamini-Hochberg corrected p-value for multiple testing.

Anthropometric indicators	Average effect[1]			Interaction with time			Effect at baseline[2]			Effect at follow-up[2]		
	β (95% CI)	p	p_{adj}[3]	β (95% CI)	p	p_{adj}[3]	β (95% CI)	p	p_{adj}[3]	β (95% CI)	p	p_{adj}[3]
**AAT**												
HAZ > −1 vs. −2 to −1	−0.11 (−0.41, 0.19)	0.49	0.96	**0.59 (0.04, 1.14)**	**0.036**	**0.046**	**−0.43 (−0.82, −0.05)**	**0.028**	**0.046**	0.16 (−0.29, 0.61)	0.63	0.96
HAZ ≤ −2 vs. −2 to −1	−0.03 (−0.36, 0.29)	0.83	0.96	0.05 (−0.58, 0.68)	0.87	0.96	−0.08 (−0.52, 0.35)	0.71	0.96	−0.03 (−0.50, 0.44)	0.90	0.96
WHZ > 0 (vs. ≤ 0)	0.02 (−0.23, 0.28)	0.85	0.96	−0.27 (−0.75, 0.20)	0.26	0.67	0.17 (−0.18, 0.52)	0.34	0.77	−0.10 (−0.44, 0.24)	0.56	0.96
**NEO**												
HAZ > −1 vs. −2 to −1	0.14 (−0.32, 0.60)	0.54	0.96	0.18 (−0.70, 1.06)	0.69	0.96	0.04 (−0.63, 0.70)	0.91	0.96	0.22 (−0.39, 0.83)	0.48	0.96
HAZ ≤ −2 vs. −2 to −1	0.01 (−0.49, 0.50)	0.98	0.98	−0.28 (−1.26, 0.70)	0.58	0.96	0.12 (−0.56, 0.80)	0.73	0.96	−0.16 (−0.87, 0.56)	0.66	0.96
WHZ > 0 (vs. ≤ 0)	−0.22 (−0.61, 0.17)	0.27	0.67	−0.41 (−1.12, 0.34)	0.28	0.67	−0.01 (−0.55, 0.53)	0.97	0.98	−0.42 (−0.95, 0.11)	0.12	0.39
**MPO**												
HAZ > −1 vs. −2 to −1	0.29 (−0.04, 0.63)	0.08	0.29	**0.68 (0.05, 1.31)**	**0.035**	0.09	−0.09 (−0.65, 0.46)	0.88	0.96	**0.59 (0.08, 1.09)**	**0.022**	0.09
HAZ ≤ −2 vs. −2 to −1	**0.31 (0.01, 0.62)**	**0.047**	0.14	0.42 (−0.29, 1.13)	0.25	0.67	0.09 (−0.41, 0.58)	0.70	0.96	**0.50 (0.02, 0.99)**	**0.043**	0.09
WHZ > 0 (vs. ≤ 0)	−0.27 (−0.56, 0.01)	0.06	0.24	**−0.59 (−1.12, −0.05)**	**0.031**	0.14	0.05 (−0.36, 0.45)	0.82	0.96	**−0.54 (−0.93, −0.15)**	**0.007**	**0.022**
